# Connexin43 Modulation of Osteoblast/Osteocyte Apoptosis: A Potential Therapeutic Target?

**DOI:** 10.1359/jbmr.0811c

**Published:** 2008-11

**Authors:** Roberto Civitelli

**Affiliations:** Division of Bone and Mineral Diseases, Departments of Internal Medicine and Cell Biology and Physiology, Washington University School of Medicine St Louis, Missouri, USA

Gap junctions are arrays of transcellular channels that allow aqueous continuity between the cytoplasms of two adjacent cells. A gap junction channel is established by docking of two “hemichannels” or connexons present on juxtaposed cells, thus forming a transcellular conduit through which ions and small molecules can diffuse from cell to cell.(1) Each connexon is composed of a hexameric array of gap junction proteins, called connexins.(2) Gap junctions are abundantly present in osteoblasts and osteocytes, and in vitro studies have shown that they can propagate signals among osteoblasts and between osteocytes and osteoblasts.(3,4) A large body of in vivo and in vitro data have established that connexins, and in particular connexin43 (Cx43), the most abundant in bone, are involved in many aspects of bone cell function, including control of osteoblastic cell proliferation, differentiation, and survival, as well as in skeletal development and postnatal bone mass acquisition.(5,6) The finding of a genetic link between the human disease oculodentodigital dysplasia and loss-of-function mutations of the Cx43 gene, *GJA1*(7,8) shows that the skeletal tissue is one of the main sites of action of Cx43. Such a link has been confirmed by mouse mutants modeling the disease.(9,10)

In addition to the ability to form gap junctions, evidence has accumulated indicating that gap junction hemichannels can exist without docking to another hemichannel, thus functioning in the guise of membrane channels of large permeability.(11) For example, Cx43 hemichannels have been shown to regulate the release of ATP and prostaglandin E_2_ (PGE_2_) in response to mechanical stimulation in osteocytes.(12) Elegant earlier work of Plotkin et al.(13) had shown that Cx43 hemichannels are intimately involved in the mechanism of action of bisphosphonates in osteoblasts and osteocytes. Such observations have given impetus to the idea that connexin may represent pharmacologic targets, because bisphosphonates are the most widely used pharmacologic agents in osteoporosis. Potent bone resorption inhibitors, bisphosphonates may also affect survival of cells of the osteoblast lineage,(13,14) although the contribution of the latter action to their therapeutic efficacy is unknown. In a series of high profile articles, Plotkin and colleagues(15,16) showed that the bisphosphonate alendronate can prevent pharmacologically induced apoptosis in osteoblasts and osteocyte-like cells and that this effect requires Cx43. Specifically, this anti-apoptotic action of alendronate is dependent on not gap junctional communication, but stimulation of src-ERK-dependent opening of Cx43 hemichannels.(17) These novel and intriguing findings are not only important for fully understanding the mechanisms of bisphosphonate action on bone remodeling, but they also disclose a potentially new direction for pharmaceutical development.

In the current issue of *JBMR*, Plotkin et al.(18) report results of a study where they tested their hypothesis in vivo in an model of corticosteroid-induced bone loss. They used mice in which they induced conditional ablation of the Cx43 gene (*Gja1*) in osteoblasts and osteocytes and showed that they can achieve effective and selective gene ablation. Although this powerful in vivo approach does not allow distinguishing between hemichannels or gap junctions as mechanisms by which Cx43 may function in this pharmacologic response, it does allow one to fully test the involvement of Cx43 in bisphosphonate action in vivo. The results of the study are bittersweet in that, although they confirm that Cx43 is involved in the anti-apoptotic effect of alendronate, they also show that neither this anti-apoptotic effect nor Cx43 is relevant for the pharmacologic effect of this bisphosphonate on prevention of bone loss induced by corticosteroid treatment. Mice genetically deficient in *Gja1* in osteoblasts and osteocytes did not exhibit, as predicted by the hypothesis, the preventative action of alendronate on prednisolone-induced apoptosis; nonetheless, they were protected from prednisolone-induced bone loss just as well as their wild-type littermates.(18) In fact, BMD was higher in both steroid-treated and untreated groups after alendronate administration, in both wild-type and mutant animals, further suggesting that the presence or the absence of Cx43 is uninfluential for responsiveness to alendronate, at least in terms of BMD. One could argue that a higher number of apoptotic cells may ultimately be detrimental for bone strength, independently of BMD, and that prevention of accumulating apoptotic cells with time may represent a positive factor. However, prolonged treatment with bisphosphonates in subjects receiving corticosteroids is not desirable, considering the suppressive effect of corticosteroids on bone formation. The complete dissociation between the anti-apoptotic effect and protection from steroid-induced bone loss emerging from the work of Plotkin et al. constitutes a strong argument against a role of anti-apoptosis and Cx43 in the pharmacologic action of alendronate. As the authors comment, the antiresorptive action of alendronate is most likely preponderant relative to other effects.

Of course, these conclusions are limited to steroid-induced bone loss, a complex condition characterized by inhibition of bone formation and relative increase of bone resorption. It would be interesting to see whether similar results occur in other forms of osteoporosis, and in particular, estrogen-dependent bone loss, a condition also associated with increased osteoblast/osteocytes apoptosis.(19) It is also possible that not all bisphosphonates have the same anti-apoptotic effect or function through Cx43-mediated mechanisms. A recent study reported that aminobisphosphonates actually increase osteoblast apoptosis, although at high concentrations, and inhibit osteoblast differentiation.(20)

The role of Cx43 for bone anabolism is much clearer. Based on earlier in vitro data showing that interference with *Gja1* expression diminishes PTH stimulation of cAMP production(21) and matrix mineralization by osteoblasts,(22) our laboratory has shown that treatment with daily doses of teriparatide (PTH fragment 1-34) results in severely attenuated increments in bone mass and reduced activation of bone formation rates in another model of conditional *Gja1* deletion, relative to wild-type mice.(23) More recently, we have also shown that stimulation of mineral apposition rate at the endocortical surface by application of a three-point bending protocol to tibiae in vivo is significantly reduced in the same mouse mutants relative to wild-type animals.(24) These results suggest that Cx43, either through gap junctions or hemichannels, or even functioning as a docking platform for signaling molecules, is important for equalizing or potentiating cell responses,(5,25) thus affecting survival, differentiation, and/or function of bone-forming cells. Therefore, despite this initial setback with bisphosphonate action, the idea of Cx43 as a pharmacologic target remains appealing. Compounds that modulate gap junction function have been produced, and one such compound is currently being developed as an antiarrhythmic agent.(26) Interestingly, this prototype compound had been shown to prevent deterioration of bone biomechanical properties in estrogen-dependent bone loss in rats.(27) The availability of several genetic models of tissue and cell-specific *Gja1* ablation or mutation makes it possible to test the potential effectiveness of gap junction modifiers in conditions of altered bone remodeling and their possible interactions with other bone active agents. The work of Plotkin et al. represents the first example of this novel therapeutic avenue, and its future potential should soon emerge.

## References

[1] Unger VM, Kumar NM, Gilula NB, Yeager M (1999). Three-dimensional structure of a recombinant gap junction membrane channel. Science.

[2] Goodenough DA, Goliger JA, Paul DL (1996). Connexins, connexons, and intercellular communication. Annu Rev Biochem.

[3] Genetos DC, Kephart CJ, Zhang Y, Yellowley CE, Donahue HJ (2007). Oscillating fluid flow activation of gap junction hemichannels induces ATP release from MLO-Y4 osteocytes. J Cell Physiol.

[4] Ishihara Y, Kamioka H, Honjo T, Ueda H, Takano-Yamamoto T, Yamashiro T (2008). Hormonal, pH, and calcium regulation of connexin 43-mediated dye transfer in osteocytes in chick calvaria. J Bone Miner Res.

[5] Stains JP, Civitelli R (2005). Gap junctions in skeletal development and function. Biochim Biophys Acta.

[6] Civitelli R (2008). Cell-cell communication in the osteoblast/osteocyte lineage. Arch Biochem Biophys.

[7] Paznekas WA, Boyadjiev SA, Shapiro RE, Daniels O, Wollnik B, Keegan CE, Innis JW, Dinulos MB, Christian C, Hannibal MC (2003). Connexin 43 (GJA1) mutations cause the pleiotropic phenotype of oculodentodigital dysplasia. Am J Hum Genet.

[8] Kjaer KW, Hansen L, Eiberg H, Leicht P, Opitz JM, Tommerup N (2004). Novel connexin 43 (GJA1) mutation causes oculo-dento-digital dysplasia with curly hair. Am J Med Genet.

[9] Dobrowolski R, Sasse P, Schrickel JW, Watkins M, Kim JS, Rackauskas M, Troatz C, Ghanem A, Tiemann K, Degen J (2008). The conditional connexin43G138R mouse mutant represents a new model of hereditary oculodentodigital dysplasia in humans. Hum Mol Genet.

[10] Flenniken AM, Osborne LR, Anderson N, Ciliberti N, Fleming C, Gittens JE, Gong XQ, Kelsey LB, Lounsbury C, Moreno L (2005). A Gja1 missense mutation in a mouse model of oculodentodigital dysplasia. Development.

[11] Goodenough DA, Paul DL (2003). Beyond the gap: functions of unpaired connexon channels. Nat Rev Mol Cell Biol.

[12] Cherian PP, Siller-Jackson AJ, Gu S, Wang X, Bonewald LF, Sprague E, Jiang JX (2005). Mechanical strain opens connexin 43 hemichannels in osteocytes: A novel mechanism for the release of prostaglandin. Molec Biol Cell.

[13] Plotkin LI, Weinstein RS, Parfitt AM, Roberson PK, Manolagas SC, Bellido T (1999). Prevention of osteocyte and osteoblast apoptosis by bisphosphonates and calcitonin. J Clin Invest.

[14] Follet H, Li J, Phipps RJ, Hui S, Condon K, Burr DB (2007). Risedronate and alendronate suppress osteocyte apoptosis following cyclic fatigue loading. Bone.

[15] Plotkin LI, Bellido T (2001). Bisphosphonate-induced, hemichannel-mediated, anti-apoptosis through the Src/ERK pathway: A gap junction-independent action of connexin43. Cell Commun Adhes.

[16] Plotkin LI, Manolagas SC, Bellido T (2002). Transduction of cell survival signals by connexin-43 hemichannels. J Biol Chem.

[17] Plotkin LI, Aguirre JI, Kousteni S, Manolagas SC, Bellido T (2005). Bisphosphonates and estrogens inhibit osteocyte apoptosis via distinct molecular mechanisms downstream of extracellular signal-regulated kinase activation. J Biol Chem.

[18] Plotkin LI, Lezcano V, Thostenson J, Weinstein RS, Manolagas SC, Bellido T (2008). Connexin 43 is required for the anti-apoptotic effect of bisphosphonates on osteocytes and osteoblasts in vivo. J Bone Miner Res.

[19] Manolagas SC (2000). Birth and death of bone cells: basic regulatory mechanisms and implications for the pathogenesis and treatment of osteoporosis. Endocr Rev.

[20] Idris AI, Rojas J, Greig IR, Van't Hof RJ, Ralston SH (2008). Aminobisphosphonates cause osteoblast apoptosis and inhibit bone nodule formation in vitro. Calcif Tissue Int.

[21] Van der Molen MA, Rubin CT, McLeod KJ, McCauley LK, Donahue HJ (1996). Gap junctional intercellular communication contributes to hormonal responsiveness in osteoblastic networks. J Biol Chem.

[22] Schiller PC, D'Ippolito G, Balkan W, Roos BA, Howard GA (2001). Gap-junctional communication mediates parathyroid hormone stimulation of mineralization in osteoblastic cultures. Bone.

[23] Chung DJ, Castro CH, Watkins M, Stains JP, Chung MY, Szejnfeld VL, Willecke K, Theis M, Civitelli R (2006). Low peak bone mass and attenuated anabolic response to parathyroid hormone in mice with an osteoblast-specific deletion of connexin43. J Cell Sci.

[24] Grimston SK, Brodt MD, Silva MJ, Civitelli R (2008). Attenuated response to in vivo mechanical loading in mice with conditional osteoblast ablation of the connexin43 gene (Gja1). J Bone Miner Res.

[25] Stains JP, Civitelli R (2005). Gap junctions regulate extracellular signal-regulated kinase signaling to affect gene transcription. Molec Biol Cell.

[26] Haugan K, Marcussen N, Kjolbye AL, Nielsen MS, Hennan JK, Petersen JS (2006). Treatment with the gap junction modifier rotigaptide (ZP123) reduces infarct size in rats with chronic myocardial infarction. J Cardiovasc Pharmacol.

[27] Jorgensen NR, Teilmann SC, Henriksen Z, Meier E, Hansen SS, Jensen JE, Sorensen OH, Petersen JS (2005). The antiarrhythmic peptide analog rotigaptide (ZP123) stimulates gap junction intercellular communication in human osteoblasts and prevents decrease in femoral trabecular bone strength in ovariectomized rats. Endocrinology.

